# Febrile illness in healthcare workers caring for Ebola virus disease patients in a high-resource setting

**DOI:** 10.2807/1560-7917.ES.2017.22.4.30449

**Published:** 2017-01-26

**Authors:** Douglas Fink, Ian Cropley, Michael Jacobs, Stephen Mepham

**Affiliations:** 1 Department of Infection, Royal Free London NHS Foundation Trust, London, United Kingdom

**Keywords:** Ebola, healthcare worker, monitoring, febrile illness

## Abstract

Ebola virus disease (EVD) patients treated in high-resource facilities are cared for by large numbers of healthcare staff. Monitoring these healthcare workers (HCWs) for any illness that may represent transmission of Ebola virus is important both for the individuals and to minimise the community risk. International policies for monitoring HCWs vary considerably and their effectiveness is unknown. Here we describe the United Kingdom (UK) experience of illness in HCWs who cared for three patients who acquired EVD in West Africa. Five of these 93 high-level isolation unit (HLIU) HCWs presented with fever within 21 days of working on the unit; one of these five presented outside of the UK. This article discusses different approaches to monitoring of HCW symptom reporting. The potential impact of these approaches on HLIU staff recruitment, including travel restrictions, is also considered. An international surveillance system enhancing collaboration between national public health authorities may assist HLIU HCW monitoring in case they travel.

## Introduction

In the recent West Africa Ebola outbreak (2013–2016), healthcare workers (HCWs) in affected countries were at particular risk of Ebola virus (EBOV) transmission and many hundreds died from EVD [1]. Only 27 medically evacuated or imported EVD cases were treated in Europe and the United States (US) during the outbreak [2], and yet despite high-resource facilities three transmissions of EBOV to HCWs occurred: two in the US and one in Spain. The exact exposures responsible for these secondary cases are not known, although in addition to providing personal care during life, the Spanish nursing assistant was involved in burial of the index case [3,4]. In 2009 the European Network of Infectious Diseases published a consensus framework for the design and operation of high-level isolation units (HLIUs) for the management of highly infectious diseases [5]. Although occupational health and safety is explicitly recognised as a high priority there is no strong evidence base for guiding monitoring of HCWs post HLIU exposure. Since early 2014, as part of an international effort, hundreds of HCWs across nine high-resource countries have cared for EVD patients and there have been numerous iterations of national guidelines concerning all aspects of EVD management including HLIU HCW monitoring [6,7]. This report reviews the recent United Kingdom (UK) experience of monitoring HLIU HCWs and managing symptomatic individuals. We consider the impact of biocontainment strategy on HCW monitoring policy and the relative strengths and weaknesses of different approaches. We reflect how best HLIU policy might protect individual and public safety without imposing exacting sanctions on a limited and often voluntary HCW population.

## Biocontainment strategy and implications for healthcare worker monitoring

In the US and most European countries, isolation units for managing EVD patients consist in negative pressure rooms where HCWs wear full-body personal protective equipment (PPE). HCWs are considered to have ‘direct contact’ with EVD patients in this setting irrespective of PPE adherence. In contrast in the UK, two HLIUs for managing cases of confirmed Hazard Group 4 viral haemorrhagic fevers (VHFs) employ a primary method of biocontainment that is quite distinct from methods used elsewhere in the world. The patient is managed within a single-bed flexible-film negative pressure isolator (Trexler isolator), which in turn is located within a negative pressure room. Care is delivered by staff wearing surgical scrubs through half suits built into the wall of the isolator itself. Early experimental pressure and virus viability studies support the clinical safety of the isolator over nearly four decades of use in the UK for management of viral haemorrhagic fevers [8].

Public Health England (PHE) defines any HCWs providing patient care in the HLIU as Category 1 contacts. Category 1 describes individuals with ‘no direct contact’ with a person with EVD. Contact by HCWs with patients while appropriately wearing the half suits of the Trexler isolator is not considered direct contact. However should Category 1 contacts record a temperature greater than 37.5 °C or develop symptoms consistent with EVD within 21 days of caring for a confirmed EVD patient they are advised to contact the physician in charge of the HLIU. This is considered passive reporting. There are no restrictions on any activities, including work and travel, of HCWs who provide care in the HLIU and remain asymptomatic.

## The recent United Kingdom experience of high-level isolation unit healthcare worker monitoring

During the recent West Africa Ebola outbreak, three cases of confirmed EVD were managed in the HLIU at the Royal Free Hospital between August 2014 and March 2015. Cumulatively this amounted to 45 patient days inside the bed isolator. Simultaneously two doctors and four nurses work 12-hour shifts in HLIU, equating to 180 doctor shifts and 360 nursing shifts during this period. In total, 46 individual doctors and 47 individual nurses undertook shifts and so 93 individuals provided direct patient care within HLIU.

Five of 93 (5.4%) HCWs who had provided direct patient care on the HLIU presented with a febrile illness within 21 days of last possible exposure to EBOV. Four of the cases were managed in the UK and one in China, where the individual was on vacation. One individual assessed in the UK had clinical features of appendicitis, which was confirmed by computed tomography scan, and did not undergo testing for EBOV infection. The other four cases had non-specific febrile illnesses and all were managed in an isolation facility with appropriate infection control precautions and were tested for EBOV infection by PCR. EBOV PCR was negative in each case, and an alternative diagnosis was subsequently confirmed in three cases (Table). One individual had an undefined febrile illness that resolved spontaneously within 48 hours.

**Table:** Healthcare workers presenting with febrile illness and admitted for assessment after caring for patients with Ebola virus disease in high-level isolation units in the United Kingdom, 2014–2015

Role on HLIU	Assessment location	EBOV real-time RT-PCR testing	Diagnosis
Doctor	RFH, London, UK	No	Appendicitis
Nurse	RFH, London, UK	Yes	Influenza A
Doctor	RFH, London, UK	Yes	Enterovirus
Doctor	RFH, London, UK	Yes	Unspecified febrile illness
Doctor	Shanghai Public Health Clinical Centre, Shanghai, China	Yes	Tonsillitis

EBOV: Ebola virus; RT-PCR: reverse transcription-PCR; HLIU: high-level isolation unit; RFH: Royal Free Hospital; UK: United Kingdom.

## Discussion

### Incidence of febrile illness in high-level isolation unit healthcare workers

The prompt diagnosis of EVD is fundamental to both individual and public health. Assessment of symptomatic HCWs who have cared for EVD patients is complex requiring prompt, coordinated, direct admission to an appropriate isolation facility, adherence to robust infection control protocols, highly trained personnel, and the expeditious sending of blood samples to a reference laboratory for EBOV testing. Our experience of this assessment pathway in the UK demonstrates that febrile illness in HCW within 21 days of last possible exposure to EBOV is not rare, occurring in 5/93 (5.4%) of HCW directly involved in patient care on HLIU. Although the reporting behaviour of HLIU HCWs might be expected to be more exacting than HCWs without exposure to Risk Group 4 viruses, this is comparable to the background rate of sick leave in National Health Service (NHS) staff of ca 4% at any time [9]. Although fever is a common symptom, reasons for NHS sick leave will include other symptoms and conditions that do not cause fever (e.g. mechanical injury). The specific incidence of febrile illness in all UK HCWs in general is not known. During winter months, encompassed by our data, a significant proportion of illness is likely to be caused by self-limiting febrile illnesses.

### Confirming alternative diagnoses

A fundamental principle of any monitoring policy in the HLIU setting remains that self-limiting febrile illnesses cannot reliably be differentiated from EVD on clinical grounds alone [10]. Apart from the case of appendicitis, which was clinically identified and did not undergo EBOV testing, the spectrum of illnesses diagnosed in the UK experience was minor and these did not require inpatient care in their own right. Besides excluding EVD, it is important to confirm an alternative diagnosis in the context of persistent fever. For a HCW population this may have separate infection control implications such as nosocomial transmission of other communicable diseases. An alternative diagnosis may also mitigate the need for repeat EBOV testing that might be indicated for high-risk exposures [11]. Deferring routine HCW influenza and other immunisation that may cause fever until more than 21 days have elapsed since last possible exposure to EBOV would seem prudent to avoid unnecessary testing. Prior to working on HLIU, routine vaccination on recruitment to the HLIU staff might be considered as a preventative measure against non-EVD febrile illness and potentially reduce the burden of EBOV testing. In time routine prophylaxis may encompass vaccination against EBOV. However this may not be possible in the context of acute clinical need and an unpredictable burden of care.

### Limitations of self-reported healthcare worker monitoring data

Divergent HCW monitoring policies exist between high-resource countries [6,7,12,13]. This variance is the probable consequence of different biocontainment strategies. In December 2015 the Centers for Disease Control and Prevention (CDC) published an update of *Interim U.S. Guidance for Monitoring and Movement of Persons with Potential Ebola Virus Exposure* [13]. This classified as ‘low (but not zero) risk’ all US-based HCWs wearing appropriate PPE caring for symptomatic EVD patients while in the ‘patient-care area’ or having any contact with a patient’s body fluids in any area. This cohort of HCWs, the vast majority of clinical and laboratory staff involved in patient care, were to be subject to ‘direct active monitoring’ for 21 days post exposure, requiring direct observation of symptoms and temperature by a public health authority at least once daily. This is opposed to ‘active monitoring’ where individuals would themselves report daily temperature. In reference to high-resource setting HCWs within the CDC guidance, ‘no identifiable risk’ described those HCWS with no exposure to the immediate patient-care area or to body fluids; ‘some risk’ described HCWs after close contact (defined as being within one metre) with a person with EVD without appropriate PPE; ‘high risk’ described HCWs after direct contact with a person with EVD or their body fluids without appropriate PPE. There were no formal restrictions on travel or work, including in healthcare settings, in the ‘low (but not zero) risk’ group. However, CDC advised discussion of plans relating to work or travel within 21 days after care of an EVD patient with local public health authorities before undertaking these activities. Further, should international travel be undertaken during this time, guidelines recommended notification of CDC and the ministry of health in the destination country with transfer of monitoring oversight [14].

In the UK reporting by HCWs is passive compared with this active and direct active reporting in the US. It is difficult to compare the risk stratification nomenclature for CDC and PHE guidelines given that they advise on different risk exposures from different containment strategies. In the sense that they both apply to HCWs undergoing routine and safe care of persons with EVD in non-endemic settings, category 1 in PHE guidelines is equivalent to ‘low (but not zero) risk identifiable risk’ in the CDC policy. However, due to the perceived added protection of the bed isolator, unlike CDC guidelines, PHE does not mandate direct active or active monitoring of symptoms or temperature for any HCWs [15].

Given that no secondary transmission of EVD occurred in the UK it is difficult to compare directly publicly available data on monitoring of HCWs in the US and our own experience. In the US, 147 contacts of two nurses diagnosed with EVD were actively monitored and 12 (8%) tested [16]. The monitored population in that case had a range of risk exposures, unlike the relatively homogenous exposures of the UK HCW cohort, and other symptoms without fever may have triggered EVD testing in this setting. Despite these significant limitations of comparing monitoring strategies, our study suggests that passive monitoring of HLIU HCWs results in similar presentation rates with 5.4% reporting febrile illness in our study. Febrile illness is likely a common phenomenon in HCWs but there are very little data regarding its diagnosis and monitoring especially in the context of emerging infectious disease outbreaks.

It is known that presenteeism, work attendance despite illness, is common in HCWs. Questionnaire-based studies of NHS staff report rates of presenteesim as high as 70% [17]. In the HLIU setting symptomatic staff may be reluctant to impose further operational demands on an already limited cohort of specialist colleagues by declaring a fever at its onset. The perceived very low risk of transmission and the high likelihood of alternative diagnoses may also influence reporting [18,19]. Therefore, despite the seriousness of EVD acquisition and clear instruction on the importance of reporting all relevant symptoms as an integral part of HLIU training, it is difficult to exclude response bias from any analysis of self-reported illness in HCWs. There is no evidence that unsupervised active monitoring reduces this compared with passive monitoring. For example, despite active monitoring and direct communication with public health authorities, it is possible that the second nurse diagnosed with EVD in the US travelled on an internal flight with febrile symptoms [20].

In the absence of clinical evidence, a more detailed understanding of HCW illness reporting behaviour and expected rates of febrile illness in these cohorts might better inform monitoring strategies.

### International collaboration

We describe one symptomatic UK HCW who was managed in conjunction with Chinese health authorities. We believe this is the first instance of EBOV PCR performed in a HCW respectively exposed and tested in two separate non-endemic countries. The individual worked in the UK HLIU, and subsequently developed febrile symptoms after arrival in China where a test for EVD was performed. Although one might argue that this represents irresponsible behaviour on the part of the HCW, there was no formal or informal requirement for the individual to alert UK health authorities before international travel within 21 days of working in HLIU. Guidelines at the European level could be helpful for a harmonised approach in the European Union in such a circumstance.

The latest CDC guidelines in early 2016 advised discussion with local authorities and notification of CDC before such travel with a view to establishing collaborative monitoring across international borders [13]. There are no published reports of any such bilateral or unilateral arrangements. Although this travel guidance may discourage HCW international travel altogether, avoiding the complexities of international public health collaboration, it does have inherent problems. Formal travel negotiations after HLIU exposure may negatively impact recruitment of HCWs. The relatively unpredictable HLIU workload means that this may be particularly relevant for those HCWs whose travel requires VISA application or other costly advanced planning. Further, the guidelines are difficult to enforce and travel restrictions or penalties for unreported travel would likely be negatively received by the healthcare community.

Our case emphasises the cross-border cooperation that highly infectious diseases may require. During the SARS outbreak, international travel was restricted for potential SARS contacts and screening strategies demanded trans-continental communication between health authorities [21]. Despite informal and formal international collaboration addressing emergency response to infectious disease threats, no systematic framework exists for monitoring and reporting contacts of persons with infectious diseases or, more particularly, HCWs across borders [22,23]. Given finite HLIU bed space and resources consideration to sharing such care between countries may be a necessary extension of such relationships. The German government has offered the services of its medical evacuation aircraft to other members of the European Union (EU) under the EU Civil Protection Mechanism [24]. Outbreaks with the potential for global spread remind us that we should continue to develop public health communication not simply across European borders but across continental borders too.

### Pragmatic monitoring

Direct active monitoring may improve the sensitivity of HCW monitoring but at significant costs. More invasive monitoring strategies, which might preclude routine work after any HLIU exposure, may negatively impact on HCW recruitment for this essential work [25].

There may be a role for pragmatic active surveillance such as monitoring via text message that has been trialled successfully in Australia representing a balance between active and passive monitoring [26]. In the event of fever, testing and home self-isolation rather than hospital admission may be appropriate to improve symptom reporting given the low risk category 1 exposure of HCW in HLIU. A formal international public health network with policy and capacity that transcends borders would empower this surveillance strategy. The principle of collective, as well as personal, responsibility would complement the remarkable contribution that diverse HCWs make to protecting global health. 
